# Numerical Simulation and Experimental Study on the Drilling Process of 7075-t6 Aerospace Aluminum Alloy

**DOI:** 10.3390/ma14030553

**Published:** 2021-01-24

**Authors:** Haitao Luo, Jia Fu, Tingke Wu, Ning Chen, Huadong Li

**Affiliations:** 1 State Key Laboratory of Robotics, Shenyang Institute of Automation, Chinese Academy of Sciences, Shenyang 110016, China; fujia@sia.cn (J.F.); tingkewu@sjtu.edu.cn (T.W.); lihuadong730@gmail.com (H.L.); 2 Institutes for Robotics and Intelligent Manufacturing, Chinese Academy of Sciences, Shenyang 110016, China; 3 Weichai Power Emission Solutions Technology Co., Ltd., Weifang 261061, China; chenning01@weichai.com

**Keywords:** numerical simulation, drilling, temperature accumulation, chip forming, tool wear

## Abstract

A finite element model for setting drilling conditions is established. The effect of feed speed and spindle speed on the drilling process was studied. In the test phase, drilling tests were conducted using three different feed speeds (60, 100, and 140 mm/min) and three different spindle speeds (800, 1000, and 1200 rpm). The correctness of the finite element model was verified by comparing the experimental and numerical simulation data. The results show that the axial force and torque increase significantly with the increase of feed speed, while the axial force and torque increase less as the spindle speed increases. The numerical simulation results show that the temperature of the cutting edge increases as the feed speed increases. Increasing the rotating speed increases the formation of chip curl. When the working conditions are high rotating speed and low feed, the tool wear is reduced, and the machining quality is better. The numerical simulation results obtained for the chip forming effect are similar to the experimental data. In addition, the simulation results show the generation of burrs. A comparison of the finite element simulation and experimental data leads to an in-depth understanding of the drilling process and ability to optimize subsequent drilling parameters, which provide reliable process parameters and technical guarantees for the successful implementation of drilling technology for space suspended ball structures.

## 1. Introduction

The aluminum alloy 7075-t6 is commonly used to manufacture aerospace components. In the connection process of most aerospace components, bolts or partial connections of holes and holes are mostly used. However, aerospace components have stringent quality requirements for drilling. Minor cracks may cause fracture failure or potential safety hazards due to sharp vibration and acceleration responses, which can cause huge losses. Therefore, the study of the drilling process parameters for the 7075-t6 aluminum alloy will help optimize it and improve drilling quality.

The drilling process was simulated through the establishment of a drilling finite element model, and then verified and corrected through experiments. Due to the time-consuming and high cost of the drilling process test, an accurate establishment of the finite element model will significantly reduce the cost of the experiment and can be applied to the early prediction of the drilling parameters of other materials. Hence, the finite element analysis method has some unique advantages.

Due to the semi-closed processing conditions for drilling, problems exist between the tool and the workpiece such as friction, high drilling temperature, and difficulty in chip removal. The drilling temperature directly affects tool wear and tool life and has an essential impact on the machining accuracy and surface quality of the workpiece. It is also one of the current main focuses of metal drilling research. The study of tool wear is not only time-consuming but also costly, so that actual studies are often replaced by numerical analysis or empirical formulas. Attanasio [1] simulated the drilling process of Inconel 718 by the finite element method. A DEFORM-3D subroutine that considers the geometry of the drill bit was developed to calculate tool wear. To save simulation time, Matsumura [2] used a 2D method to study and analyze energy and chip flow during drilling.

With the development of computer technology, computer-aided design has been widely used in the field of drilling with remarkable results. Some physical phenomena unsuitable for observation in the drilling process can be clearly and intuitively determined using finite element simulation, such as changes in residual stress and strain, drilling temperatures, and axial forces. Chatterjee et al. [3] optimized the drilling process conditions through algorithms and analyzed the drilling hole diameter by the finite element method in the DEFORM software. The drilling temperature influences the roundness and cylindricity of the hole and can verify the roundness error. Ucun [4] studied the drilling force and moment of aluminum alloy materials and confirmed the values by experiments and simulations. The results of the simulation and test data are 80–90% consistent.

Drilling is a semi-closed cutting process. A fundamental problem in drilling is that the sizeable axial force on the drill bit shortens the drill bit’s service life. Wu [5] established an equivalent drilling model, considering the effect of feed rate. It is used to predict the drilling force distribution on the main cutting edge and chisel edge of the drill bit. Nouari [6] studied the change of wear mechanism in the drilling process versus the change of cutting conditions and bit type for the Al2024 aluminum alloy. The results show that the HSS drill bit is not useful for the drilling process of Al2024 aluminum alloy under dry cutting conditions. The geometry of the cutting tool edge directly affects the chip formation mechanism in machining. Orthogonal cutting experiments were carried out on the Ti6Al4V titanium alloy by Oliaei [7]. The effects of combined edge formation on the axial force and surface roughness were studied. The results show that there is a close relationship between the minimum uncut chip thickness and the average roughness of the machined surface.

In drilling process and wear mechanics, Wang [8] measured the fiction coefficient of PDC bearing, especially under drilling fluid environment to provide support for the design of the downhole tool where the bearing friction coefficient obviously affects the feasibility and agility of the tools. Kumar [9] introduced a drill vibration index (DVI) as an indicator of vibration severity of the drill machine and studied its relationship with ROP and average mean partial size. Khanna [10] compared drilling performances of carbon fiber reinforced plastic (CFRP) composites under dry and cryogenic environment.

Drilling temperature has an important influence on drilling quality. Feito [11] calculated the thermal distortion of the tool and workpiece using the finite element model, which can be used to calculate the relationship between the diameter and depth of the borehole. Due to the complex geometry of the bit, the material being drilled varies significantly in strain, strain rate, and cutting temperature. Patne [12] developed a comprehensive finite element model by combining the cutting edge radius of the tool followed by evaluation of the temperature distribution in drilling via consideration of the heat distribution factor. Li [13] used the reverse heat transfer method to study the distribution of tool temperature during pure titanium drilling. The temperature distribution of the drill bit was solved by the finite element method, and local verification was carried out by experimental measurement. Excellent consistency was demonstrated. Siddharth [14] studied drilling under dry and low-temperature conditions by analyzing the influence of cutting speed and feed rate on the axial force, delamination, and surface roughness. The research showed that, as temperature decreased, the stratification factor and surface roughness both showed a downward trend, while the axial force showed an upward trend.

The formation of chips also affects tool wear during drilling. Pervaiz [15] showed that chip accumulation reduces the surface quality and tool life and directly depends on the heat generated by the tool surface in the cutting area. However, the geometric parameters of drilling tools are also critical in the drilling process, because they directly affect the surface integrity of the workpiece [16] and the cutting force [17]. 

The finite element model is modified according to the axial force and torque measured by orthogonal experiment with multiple parameters, which effectively improves the correctness of the finite element simulation. It provides a reference for the subsequent optimization of drilling quality and drilling parameters during the actual drilling process. Numerical simulation of processing parameters by finite element simulation technology can effectively analyze the processing and molding process, reduce the test cost, and shorten the product development cycle.

Due to the complexity of drilling tools and machining processes, the equipment for aerospace applications requires extremely high drilling quality and precision. Therefore, the study of drilling technology will help optimize drilling parameters. Dynamic modeling methods and analysis of chip, mechanical properties, and temperatures under complex working conditions need further investigation.

## 2. Finite Element Simulation of Drilling

### 2.1. Application Model for the Drilling Structure

**Figure 1:**
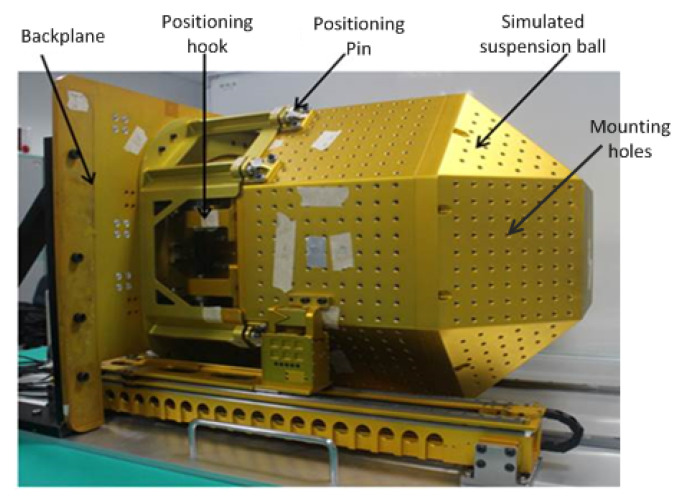
Space station suspension ball structure.

Different from traditional drilling technology, the objective of drilling for aerospace applications is usually fabrication of high-density hole structural parts such as skin, backplane, and truss, as shown in Figure 1. The backplane and space station suspension ball are made of aluminum alloy, which is generally 7075-t6 aluminum alloy with a thickness of 4–10 mm, to reduce the weight of aerospace equipment. The space station suspension ball is provided with high-density mounting holes, which are mainly used for positioning and mounting optical targets and devices. Therefore, the requirement for dimensional accuracy and quality of holes is high. Considering the complexity of thin-walled structural parts in aerospace equipment and the application of drilling simulation research, this paper studies the variation of parameters such as axial force, torque, and drilling temperature in the drilling process based on single-board processing research, as well as the critical processing parameter characteristics under different drilling parameters. In this case, to reduce the calculation time, only the part of the workpiece slightly larger than the processing area is selected.

Space suspended ball structure will be used in space in the future, so higher requirements have been established for drilling accuracy, quality, and structural strength for its panels. Panel drilling on the space suspended ball structure is currently accomplished based on experience, but it is challenging to meet the drilling quality specifications. Therefore, research on the drilling process parameters is of great significance for optimizing drilling quality. The research results will be applied to the drilling process of the space suspended ball structure in the future.

### 2.2. Establishment of the Finite Element Model

This study uses DEFORM-3D finite element software (v10.2, Scientific Forming Technologies Corporation (SFTC), Columbus, OH, USA) to simulate the drilling process that can be used in metal forming, machining, and heat treatment processes. The aluminum workpiece material is 7075-t6, and the drill bit (Dongguan Xuhua Tool Technology Co., Ltd, Dongguan, China) material is WC (wolfram carbide). Its geometric shape and drilling parameters are shown in Table 1. Workpiece materials and drill bits are loaded from the DEFORM library, and their thermo-mechanical properties are shown in Table 2. A fine mesh of 0.15 mm is placed between the point angle of the drill bit and the drilling area because severe deformation will occur during drilling in this area. Hence, the software uses adaptive meshing technology.

The workpiece is plastic, and the drill bit is rigid. Drill forming is a problem of a sizeable non-linear deformation, and also has the characteristics of continuity and dynamics. The coordinates of the element nodes are modified step-by-step as the deformation of materials occurs in the drilling area. Some elements are extruded or distorted due to non-uniform deformation, which seriously affects the accuracy of the solution, and even can lead to serious distortion or non-convergence of the calculation results due to the distortion and degradation of the mesh. To ensure the accuracy of calculations and prevent the occurrence of distorted elements, the mesh must be re-divided over time in the finite element simulation.

The number of simulation steps represents the number of iterations in the simulation solution needed to predict the chip geometry and thermal solution at a steady state. Lagrange transient analysis has the advantage of simplifying the transient drilling process and the scheme of continuous chip formation. The drilling process is a continually changing process, which can only be processed at times according to the transient process. Therefore, transient analysis is continuously operated to complete final drilling.

### 2.3. Boundary Conditions

**Figure 2:**
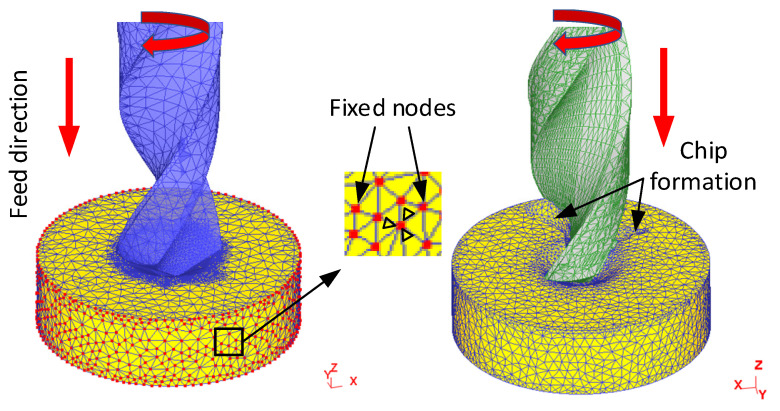
Constraints of the simulation.

In the boundary condition, the speed of the side surface and the bottom surface of the workpiece in the x, y, and z directions are set to zero. The purpose of these settings is to ensure that the workpiece is stationary, and the cutter makes feed movement in the z-direction and rotates around its axis. The constraint conditions for the simulation are shown in Figure 2.

In the simulation process, according to Ucun [4], the ambient temperature is set to 20 °C, the convection coefficient is 0.02 N/s/mm/°C, the friction type is defined as shear friction, the friction coefficient is set to 0.55, and the heat conduction coefficient is set to 45 N/s/mm/°C. The conjugate gradient method is used to solve the problem. Ucun studied the drilling force and torque of aluminum alloy material and verified it by experiment and simulation. The results show that the consistency between simulation and test data is 80–90%. Therefore, in this paper, the simulation parameters are referred to carry out experiments, and the simulation results are in good agreement with the actual situation. The iterative method used is the direct iteration method. The empirical formula for the Usui [18] model is used for the tool wear model.

### 2.4. The Flow Stress of Materials

Drilling is a process of plastic deformation and chip separation from the workpiece. A large amount of heat is generated during metal drilling, which is followed by large plastic deformation. According to Hossein [19], chip formation is simulated at high temperatures and high strain rates. The accurate material constitutive model is the key to simulate successfully metal drilling.

**Figure 3:**
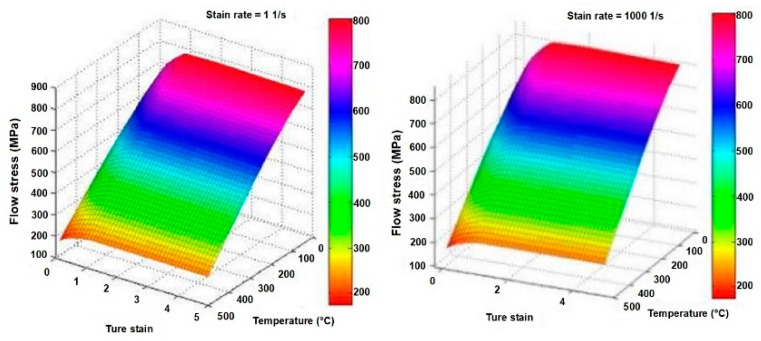
Flow stress curve for the 7075-t6 aluminum alloy.

The material constitutive relationship in the software database can only reflect the authenticity of a drilling simulation to some extent. The flow stress data over the temperature range of 20–500 °C and a strain range of 0.05–5 are collected to obtain a more accurate simulation. Some sress–strain relationships are shown in Figure 3.

The Johnson–Cook material model simulates the flow stress of the workpiece. According to Johnson et al. [20], the calculation formula for flow stress is Equation (1).

(1) $$\sigma =\left(A+B\varepsilon^{n}\right)\left(1+C\ln\left(\frac{\dot{\varepsilon }}{{\dot{\varepsilon }}_{0}}\right)\right)\left(1-{\left(\frac{T-T_{r}}{T_{m}-T_{r}}\right)}^{m}\right)$$

where *A* is the yield stress, *B* is the strain hardening coefficient, *m* is the thermal softening coefficient, *n* is the strain hardening index, *C* is the strain rate sensitivity parameter, *σ* is the flow stress, *ε* is the equivalent plastic strain, $\dot{\varepsilon }$ is the strain rate, ${\dot{\varepsilon }}_{0}$ is the reference strain rate, *T* is the temperature, *T_r_* is the room temperature, and *T_m_* is the melting temperature. The Johnson–Cook material constants are listed in Table 3 (see [21]).

### 2.5. Friction Model

Friction plays a vital role in metal drilling. It defines the relationship between friction on the drilling tool and interface stress. In metal cutting, according to Ceretti [22] and Arrazola [23], the value is between 0.5 and 0.6. In this study, the friction coefficient of 0.55 is selected as the value of μ. Comparing the simulation result with the experiment shows that the best effect occurs when Equation (2) is followed.

(2) $$\tau =\mu \sigma_{p}$$

### 2.6. Damage Model

During drilling, material damage will inevitably occur, which leads to chip generation and detachment. Therefore, the establishment of a damage model has an important impact on simulation accuracy. Hence, the Oyane criterion as a function of the mean stress ($\sigma_{m}$) and the effective stress ($\bar{\sigma }$) was used (Equation (3) [24]).

(3) $$K_{2}=\int_{0}^{{\bar{\varepsilon }}_{c}}\left(1+\frac{1}{K_{1}}\frac{\sigma_{m}}{\bar{\sigma }}\right)d\bar{\varepsilon }$$

where ${\bar{\varepsilon }}_{c}$, $\bar{\varepsilon }$, *K*_1_, and *K*_2_ are the fracture strain, equivalent strain, model coefficient, and critical damage value, respectively.

### 2.7. Heat Transfer Model

The rotary extrusion of the tool causes plastic deformation of the material and heat generation during plastic deformation (see Equation (4)).

(4) $${\dot{q}}_{p}=\frac{f{\dot{w}}_{p}}{\rho }$$

where ${\dot{w}}_{p}$, ρ, and *f* are, respectively, the plastic work rate, density, and the coefficient of plastic work conversion into heat. The generated heat causes the temperature of the drilling area to rise. According to Hossein [19], a small portion of the heat is transferred to the environment through thermal convection.

During the drilling process, chips are generated continuously, which is the heat source generated by friction at the tool–chip interface. Equation (5) represents the heat generated by friction.

(5) $$\dot{q}=n\tau v_{s}$$

where *n* is the friction work conversion coefficient, τ is the friction stress, and $v_{s}$ is the sliding speed. According to Haddag [25], heat is transferred from the friction interface to the tool, causing the tool temperature to rise. Excessive temperatures can increase tool wear.

### 2.8. Tool Wear Mode

Since drilling is a continuous process, according to Yen [26], the Usui model is applicable. This model is represented by Equation (6).

(6) $$w=\int apVe^{-\frac{b}{T}}dt$$

In the formula, *p* is positive pressure; *T* is the absolute temperature of the cutter face; *a* and *b* are constants, which are determined by the drilling parameters and materials; and *V* is the sliding speed of the workpiece material relative to the tool.

## 3. Test and Data Acquisition

**Figure 4:**
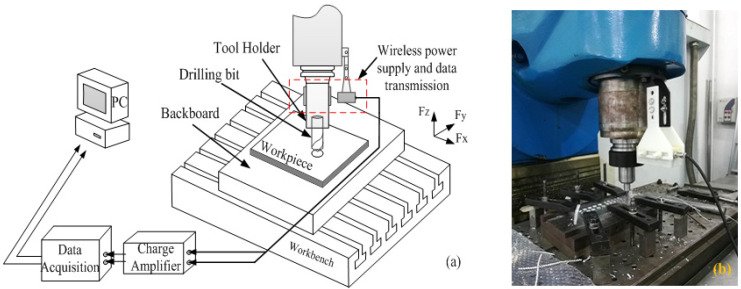
Data acquisition process: (**a**) data acquisition schematic diagram; and (**b**) data acquisition experimental set-up.

The test and data acquisition methods are shown in Figure 4a. The drilling process tests have the same drilling depths, workpiece thicknesses, and clamping conditions. The diameter of the drill is 10 mm, and the material is W6. The material in the simulation process is consistent with the experimental material. The spindle speed and feed speed are selected as variable parameters in this study. The maximum milling speed is 2200 rpm. Through the electromagnetic coupling technology, the axial force and torque data of the tool drilling process are collected in real time. 

Electromagnetic coupling technology is used to collect the axial force and torque data of the tool drilling process in real-time. Most scholars use the method of setting the mechanical sensor at the bottom of the workpiece for data acquisition. However, when the drilling center deviates from the sensor center, the accuracy of the test method is significantly reduced.

In the electromagnetic coupled resonant wireless transmission system, the first side uses the amplitude shift keying (ASK) method to load data signals into the power transmission channel and transmits them synchronously. On the secondary side, the signal is extracted by non-coherent demodulation, and the coil restores the extracted electric energy. The schematic diagram of the acquisition method is shown in Figure 4a. The synchronous transmission method of electrical energy and signals can reduce the transmission inversion links and improve forward signal transmission accuracy.

## 4. Results and Discussion

### 4.1. Axial Force and Torque

In the drilling process, the friction between drill bit and chip, as well as between drill bit and workpiece, produces drilling resistance. Due to the influence of drilling force and torque, the workpiece will produce processing deformation. A large processing deformation will significantly affect the quality and performance of the workpiece. It needs to be analyzed and minimized. Therefore, it is necessary to analyze and predict the axial force and torque in the drilling process.

**Figure 5:**
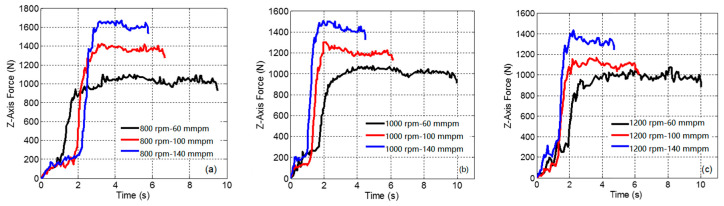
Axial force difference at different feed rates. (**a**) 800 rpm; (**b**) 1000 rpm; (**c**) 1200 rpm.

**Figure 6:**
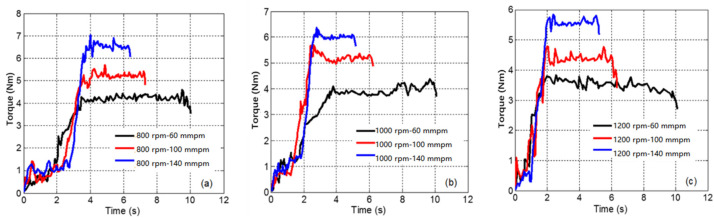
Spindle torque differences at different feed rates. (**a**) 800 rpm; (**b**) 1000 rpm; (**c**) 1200 rpm.

According to the curve of torque and axial force in the drilling process shown in Figure 5 and Figure 6, the drilling process can be roughly divided into an initial plunging stage and a steady drilling stage. In the initial plunging stage, the axial force and torque show a non-linear upward trend as the cutting depth increases due to the influence of cutting resistance and friction. When all the blade angles are found in the simulation and experiment. As the drilling reaches a stable state, these enter the material, due to the influence of chip fracture, the axial force and torque curves fluctuate, and similar load c variation of the axial force and torque on the bit tends to be small, but there are still a few fluctuations.

**Figure 7:**
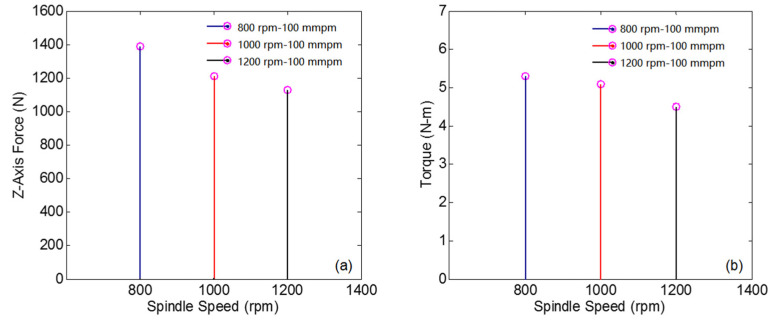
The influence of spindle speed: (**a**) the impact of spindle speed on the axial force; and (**b**) the influence of spindle speed on torque.

As shown in Figure 7, the axial force and torque at different speeds are compared and analyzed when the feed speed is 100 mm/min. That is to say, this experiment is only to obtain the influence of spindle speed, so it is necessary to keep the feed speed unchanged, and the influence of feed speed on axial force and torque is shown in Section 4.2. When the spindle speed increases from 800 to 1200 rpm, the axial force and torque decrease, but the influence is relatively small. According to Amini [27], the cutting force decreases as the spindle speed increases, which is due to the reduction of chip accumulation and friction on the cutting surface. In contrast, the influence of feed speed on the axial force and torque is more significant.

### 4.2. Finite Element Model Verification

**Figure 8:**
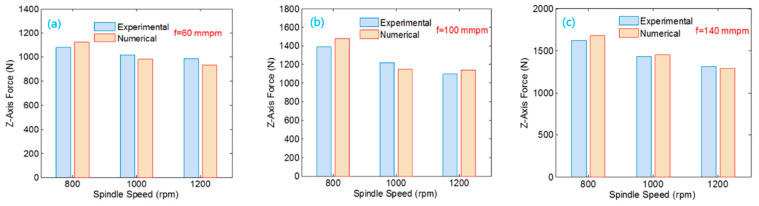
Data comparison for different feed speeds: (**a**) feed speed is 60 mm/min; (**b**) feed speed is 100 mm/min; and (**c**) feed speed is 140 mm/min.

The experimental and numerical simulation data for the axial force are compared, and the two datasets are similar. When the rotational speed is constant, the axial force increases as the feed speed increases. When the feed speed is constant, the axial force decreases as the rotational speed increases. When the rotation speed is 800 rpm, and the feed speed is 100 mm/min, the maximum error is 3.25%. Figure 8a shows that the spindle speed has a relatively small influence on the axial force when the feed rate is low.

**Figure 9:**
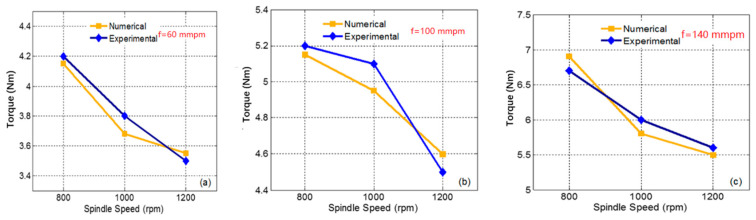
Torque comparison of different feed rates: (**a**) feed speed is 60 mm/min; (**b**) feed speed is 100 mm/min; and (**c**) feed speed is 140 mm/min.

As shown in Figure 9, the overall trend of the numerical simulation results is consistent with the experimental tests. Under the same feed speeds, the spindle torque decreases with an increase in spindle speed, and the overall test results of torque are slightly higher than the numerical simulation data. A comparison of the results shows that the simulation and experimental data are consistent.

When the rotational speed is 1000 rpm, and the feed speed is 140 mm/min, the maximum error of the torque is 4.16%. The simulation data and test data maintain a notable downward trend. By comparing the axial force and torque of the experiment and simulation process, the numerical simulation data are reliable, the simulation error is within an acceptable range, and the feasibility of the finite element model is verified.

### 4.3. Temperature Analysis during Drilling

**Figure 10:**
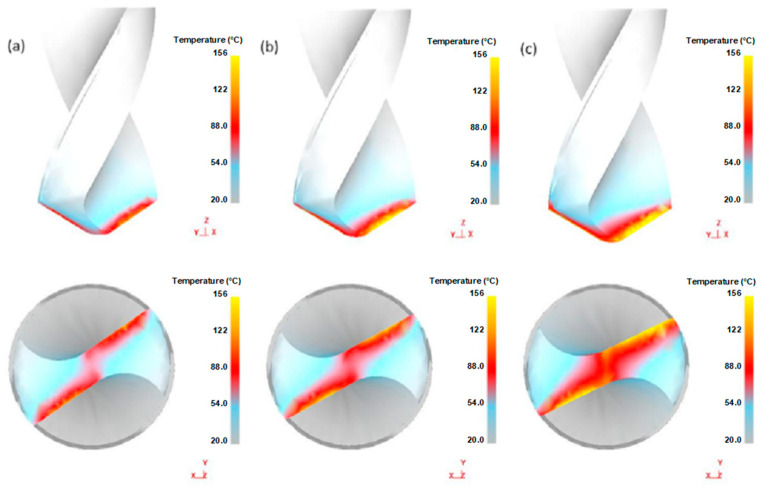
Tool temperature differences at different feed speeds (spindle speed is 1000 rpm): (**a**) the temperature when feeding at 60 mm/min; (**b**) the temperature when feeding at 100 mm/min; and (**c**) the tool temperature when feeding 140 mm/min.

Through the axial force and torque data used to verify the correctness of the finite element model and modify the finite element model, it was determined that the temperature field in the drilling process has an important impact on the forming quality. Due to the limited space occupied by the drill bit, collecting the temperature of the drill bit area through embedded thermocouples does not work, so the temperature of the drill bit is simulated through the modified finite element model, as shown in Figure 10. As the feed rate increases, the temperature of the bit increases significantly. The rise in feed rate leads to an increase in cutting thickness. As shown in Figure 5 and Figure 6, both the axial force and the torque increase significantly. At the same time, the friction resistance of the interface increases, resulting in a higher temperature of the contact area.

In the process of drilling, a large amount of heat is generated due to friction at the tool–chip interface and plastic deformation. The heat generated during drilling is transferred to the chips, the workpiece, the tool, and the surrounding medium. The drilling temperature of each part is not only related to the heat generated but also related to the thermodynamic characteristics and heat source characteristics of the part. There are three primary sources of heat during the drilling process:

(1) shear deformation heat generated by the shear zone;
(2) friction heat generated by contact between the utter and chip; and
(3) friction heat generated in the contact area of cutter and workpiece.

The heat flux on the cutter is mainly friction and conduction heat. In addition, there are thermal convection and thermal radiation between the tool–workpiece system and the surrounding medium.

Figure 10 shows the temperature distribution of the drill bit. The maximum temperature of the tool is located in a small area at the junction of the rake face and the flank face. During the drilling process, the flank face of the tool is enclosed in the workpiece, and the rake face is in contact with the workpiece due to rotation, and part of the heat is transferred into the workpiece. The junction between the rack face and the flank face is in a relatively enclosed state with little heat dissipation and much heat accumulation. Therefore, the temperature here is higher and has better consistency with the actual processing temperature.

**Figure 11:**
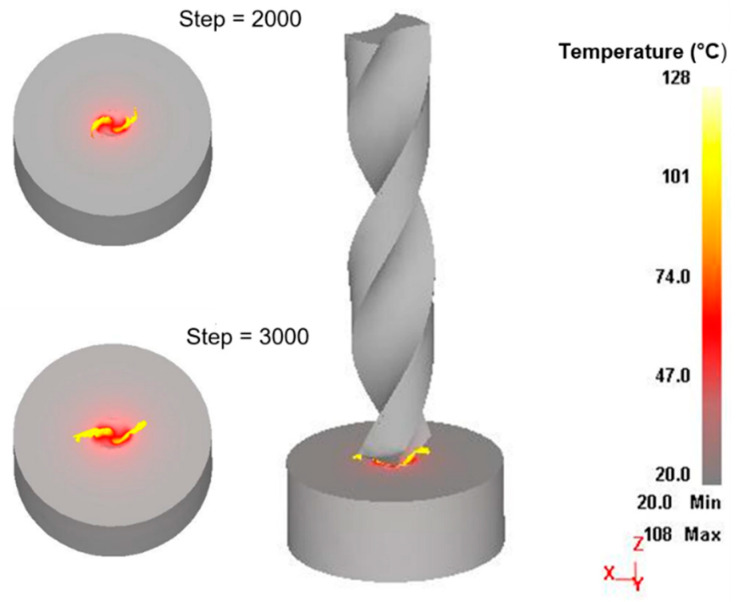
Temperature transfer during drilling.

The thermal history of drilling is an essential feature in the drilling process because most of the mechanical energy required for machining is converted into heat, which is then distributed to tools, chips, and workpieces. The temperature of the drill bit directly affects the service life of the tool. Drilling heat plays a vital role in the drilling process. Meanwhile, drilling temperature has an important influence on drilling torque, tool wear, and chip formation. Drilling heat is mainly caused by large deformation and friction of the metal. As shown in Figure 11, chips take away drilling heat.

**Figure 12:**
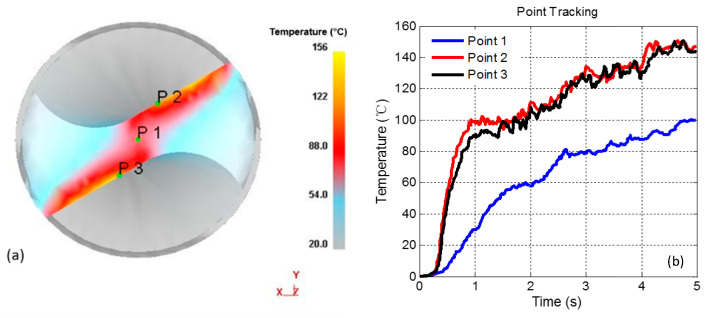
Particle tracking of tool temperature. (**a**) Three particle points marked on the drilling edge; (**b**) Temperature change of the three particle points.

The temperature of the tool is tracked while rotating at 1000 rpm and a feed rate of 100 mm/min. The purpose of this paper is to study the distribution and variation of bit surface temperature in the process of drilling. Only representative particles need to be selected for research, and not too many points need to be selected. In the same way, we only need to take the representative test from many tests for analysis. As shown in Figure 12a, three particle points are marked on the drilling edge: p1 is set at the center of the chisel edge of the drill bit and p2 and p3 are placed on the two main drilling edges. The temperature change of the three particle points is tracked. As shown in the temperature curve for the three points in Figure 12b, the temperature rises rapidly in the initial plunging stage, and the temperature changes in the stable state of drilling are relatively small. The temperatures at p2 and p3 are the same, and the changing trend is also similar. The overall change trend for the p1 particle set on the chisel edge is similar to p2 and p3, but the temperature there is slightly lower than on the main drilling edge because the main drilling operation does not take place at P1, but at P2 and P3. P1 is mainly generated by friction heat, so the temperature rise caused by drilling heat is more obvious at P2 and P3 than at P1, which is also in line with the actual situation. The side proves that our virtual experiment is consistent with the actual situation, and more specifically reveals the distribution and variation law of bit surface temperature in the drilling process Wear law.

**Figure 13:**
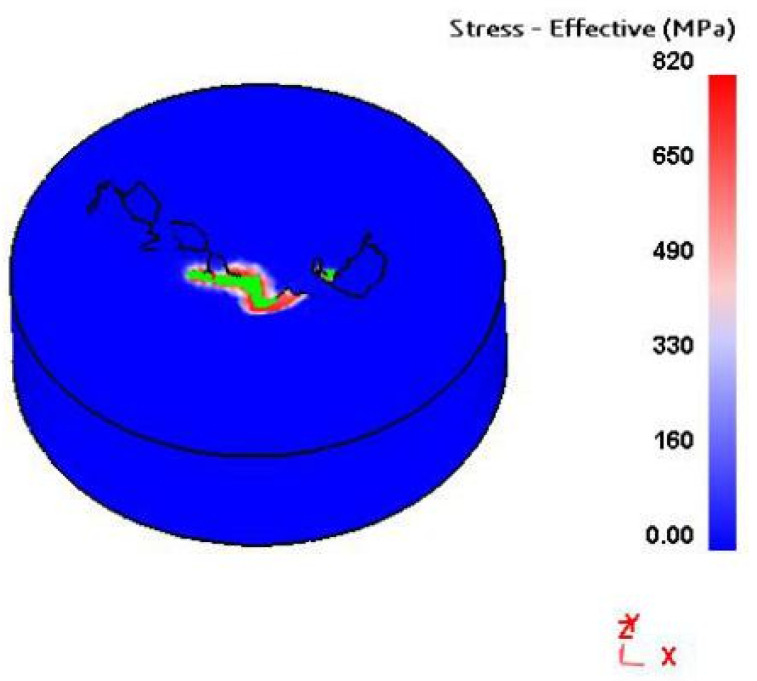
Effective stress distribution.

Combined with the analysis of the effective stress diagram of the workpiece in Figure 13, it can be seen that the effective stress is most significant at the beginning of chip formation in the drilling area, which produces higher deformation energy. At the beginning of chip formation, the friction between the drilling edge and the workpiece is more significant, and the heat generated by friction is also concentrated in the drilling area. Deformation energy and heat generated by friction cause the chip temperature to rise.

### 4.4. Tool Wear

**Figure 14:**
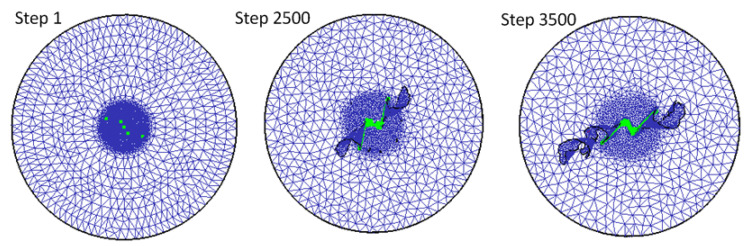
The contact area of the tool and the workpiece.

The direct contact area of the tool is the load area of the tool. During the drilling process, the rake face contacts the workpiece. As the tool is fed, chips are continuously generated and discharged along the chip groove. According to Gómez [28], chips adhering to the drill groove will block the exit path of the chips, resulting in a significant increase in axial force, torque, and tool wear. From the contact area diagram in Figure 14, the chisel edge of the drill bit and the two main drilling edges contact the workpiece and form a “z” shape. As the drilling continues, the “z” shape gradually increases until reaching the maximum working diameter of the cutter. The contact area of drilling further explains why the main drilling edge wears quickly.

The simulation data show that the wear of the drill bit increases gradually as the drilling time increases. Compared with the low feed rate, the drilling edge wear at a high feed rate is greater. When the feed rate increases, the drilling thickness increases, the axial force and torque on the drill bit increases, the shear stress of the material increases, and the wear of the drill bit increases. Compared with the working conditions at high rotating speeds, tool wear is more significant at low rotating rates. When the spindle speed increases, the friction degree between the drill bit and the workpiece increases, but the temperature in the drilling area of the workpiece increases, which aids the drilling process.

**Figure 15:**
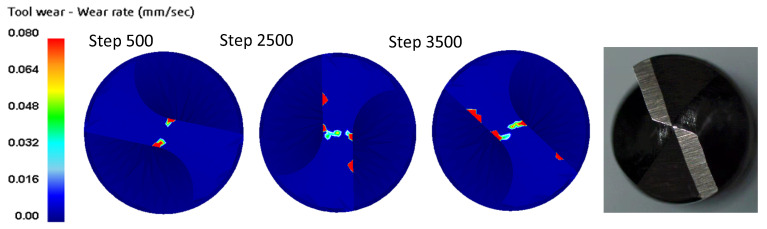
Simulated and experimental drill wear.

In general, the wear rate of the tooth surface increases as the feed speed increases, while the wear rate of the tooth surface decreases as the rotational speed increases. In addition, Figure 15 shows the wear distribution of the tool surface in the test and simulation. When the simulation step increases from 500 to 3500, wear occurs in the contact area between the tool and the workpiece, and it is also distributed along the drilling edge.

### 4.5. Burr Generation

**Figure 16:**
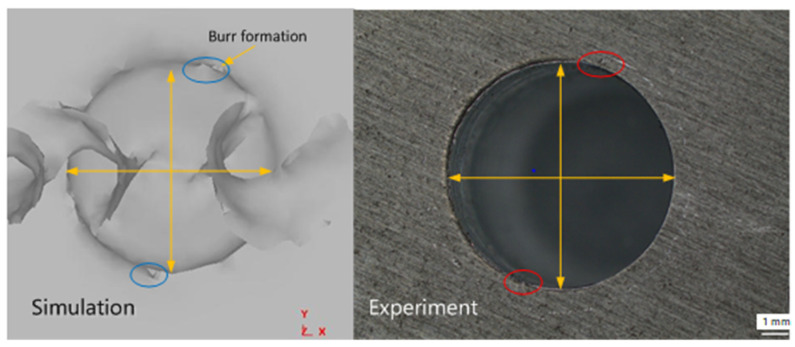
Drilling formation and burr generation (spindle speed is 800 rpm and feed speed is 140 mm/min).

Due to the reduced processing rigidity of thin-walled parts, the lower strength of the workpiece is more prominent, so drilling burrs occur quickly. According to Wang [29], some materials adhere to the surface of the drill bit, making the resulting surface quality worse, and the rest of the material is discharged as debris. In addition, due to the high toughness and elongation of the aluminum alloy, this material characteristic makes the aluminum alloy workpieces easy to produce larger drilling burrs in the processing process. As shown in Figure 16, the numerical simulation results are similar to the test results and have sound hole forming conditions, while the numerical simulation results also show the generation of burrs.

The rotating speed improves the surface quality significantly resulting from better shearing of the tool at higher rotating speeds. This result is contrary to the effect observed for feed rate, where the surface quality decreases as the feed rate increases. Due to the increase in feed rate, chip curling increases. Generally speaking, part of the workpiece material adheres to the drill bit during drilling, and some parts of the adhered material are separated from the drill bit as drilling progresses. In the drilling process, adhesion and separation occur continuously, and the drilling quality is better under high rotating speed and low feed conditions.

### 4.6. Chip Geometry and Material Damage

**Figure 17:**
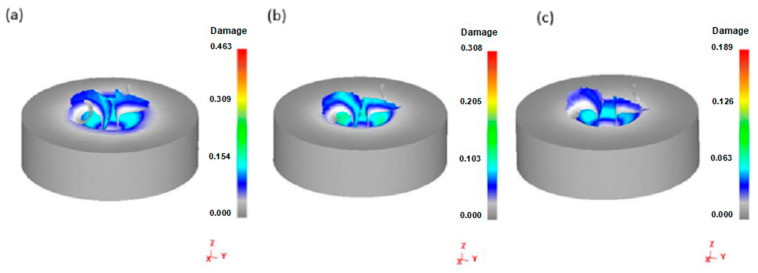
The damage value of materials at a spindle speed of 1000 rpm: (**a**) at a feeding rate of 60 mm/min; (**b**) at a feeding rate of 100 mm/min; and (**c**) at a feeding rate of 140 mm/min.

Workpiece material damage values should be evaluated first when studying chip geometry. This damage should be understood because, in addition to the drilling geometry and the workpiece material, this value directly defines the geometry of the deformed chips in the simulation. Therefore, damage values at different feed rates are shown in Figure 17. The results show that the damage value changes significantly with the change in feed rate. In addition, when the feed speed is the lowest, the damage value is the highest. For the highest feed rate, the damage value is the lowest. This result can be explained by analyzing the rake angle of the drilling process. During the drilling process, the rake angle of the drill bit changes along the length of the cutting edge, with the lowest at the chisel edge. The rake angle is calculated by Equation (7):

(7) $$\tan\alpha =\frac{r_{x}\tan\omega }{R\sin\varphi }$$

where *R* is the radius of the drill bit, *r_x_* is the radius of the selected point on the length of the drilling edge, ω is the helix angle, and φ is half of the drill point angle.

In general, the value of the rake angle is different from its calculated value during drilling. In addition to the rotary motion of the drill bit, the feed motion makes every point on the length of the drilling edge pass through a spiral path, and the pitch of the spiral track is equal to the feed value [30]. Therefore, the rake angle ($\alpha_{f}$) in the drilling process is obtained from Equation (8):

(8) $$\alpha_{f}=\alpha +\tan^{-1}\frac{f}{\pi D}$$

where *f* and *D* are the feed rate and the drilling diameter, respectively, and α is the rake angle in the drilling process. According to Equation (8), the decrease in feed speed leads to a decrease in tool rake angle. When the rake angle of the bit decreases, chip curl increases during drilling. According to Mohammad [31], the tool needs to apply a significant bending moment at the root of the chip deformation to make the chip break. Figure 17 shows the correct choice of the damage model in this study in which a spiral chip similar to that observed under experimental conditions is produced in the simulation.

In the drilling process, chip formation, heat generation, chip friction, and drilling curls all affect each other. The drilling process increases the friction force at the interface, thus causing the temperature and heat in this area to rise rapidly. High temperature promotes the formation of chip curling. As shown in Figure 10, as the feed rate increases, the temperature distribution of the surface of the drill bit increases, and the high temperature concentrates on the drilling edge.

**Figure 18:**
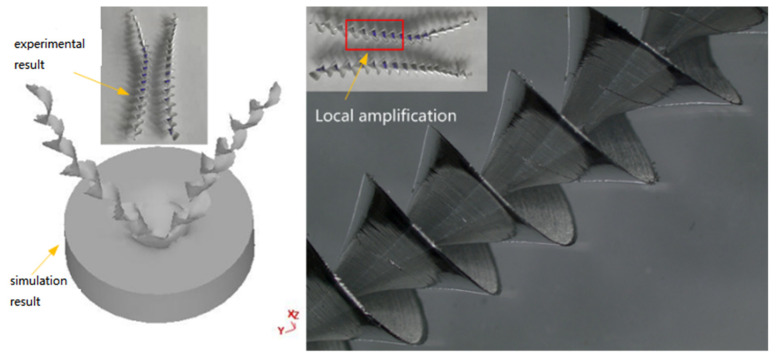
Formation and comparison of chips during experiment and simulation.

Figure 18 shows that the numerical simulation is similar to the chip formation shape in the test results. As shown in the chip micrographs in Figure 18, when the drilling quality is good, the chip surface is smoother, and burrs and flash are fewer. The chip forming process has an important influence on the quality of drilling, so it is essential to choose a reasonable damage model in the simulation process to simulate the real conditions of drilling. The test results verify that the material damage model can predict chip forming well.

The validity of the finite element model is confirmed through the comparison of axial force and torque data. Additional research on drilling process temperature, tool wear, and chip forming will improve the understanding of the drilling process and expertly guide the selection of test parameters. Using finite element software to simulate the drilling process can assist engineers in the analysis and understanding of the material forming process. It can reduce the use of expensive drilling production materials and labor costs, shorten the product research and development cycle, and improve enterprise benefits.

Research on the drilling process is of considerable significance for the application of porous structural members. It is easier to understand the drilling process by analyzing simple workpieces. At the same time, the study of drilling process temperature, axial force, torque, tool wear, and chip forming has guiding significance for optimizing drilling parameters. Due to the high precision and quality requirements of the mounting holes on the space suspended spherical plate, the processing deformation and structural damage of the panel needs to be reduced. The existing processing parameters of the space suspended spherical plate are mostly based on experience, but the processing quality does not reach the expected target. In the future, the results of the parameters study of simple workpieces can be applied to the drilling process of the space suspended sphere structure.

## 5. Conclusions

In this study, the experiments and numerical simulation of 7075-t6 drilling were studied. The effects of drilling parameters on torque, axial force, temperature, stress, tool wear, and chip formation were determined. Through this research, the following conclusions are drawn.

(1) The comparison of numerical simulation and experimental data is consistent, and the reliability and predictability of the finite element model are verified.
(2) At the same rotating speed, the axial force and torque of the drill bit increase significantly as the feed speed of the drill bit increase from 60 to 140 mm/min. As the feed speed increases, the thickness of uncut chips increases and chip formation becomes more difficult.
(3) Under the same feed speed conditions, as the rotation speed of the drill bit increases from 800 to 1200 rpm, the axial force and torque of the drill bit decrease. Increasing the rotating speed increases the friction between the drill bit and the material, thus accelerating the temperature rise and softening the material plastically, whihch, in turn, reduces the axial force and torque.
(4) The numerical simulation of the tool temperature field shows that the temperature at the cross edge is lower than the main cutting edge. When the load on the tool is smaller, the tool wear is lower at high speed, and at low feed the drilling formation quality is better.
(5) The numerical simulation results of the chip forming process are similar to the experimental results, and the numerical simulation results also show the formation of burrs.
(6) The finite element model developed in this work can accurately simulate drilling parameters. The analysis results of the finite element simulation of the drilling process can be used for the optimization of subsequent drilling process parameters. The finite element simulation of chips and burrs is helpful to analyze further and study the formation mechanism of chips and burrs in drilling.

## Figures and Tables

**Table 1:** Drill bit geometry and cutting parameters used in the simulation.

Tool Parameters	Value
Drill diameter (mm)	10 mm
Point angle (°)	118°
Helix angle (°)	30°
Feed speed (mm/min)	60, 100, 140 mm/min
Spindle speed (rpm)	800, 1000, 1200 rpm

**Table 2:** Thermo-mechanical properties of AL7075 and WC.

Properties	AL7075	WC
Young’s modulus (MPa)	71,700	630,000
Density (kg/m^3^)	2810	12,800
Poisson’s ratio	0.33	0.22
Heat capacity (J/(kg·°C))	960	226
Conductivity (W/(m·°C))	173	44.6

**Table 3:** Material constants for Al 7075-t6.

A (MPa)	B (MPa)	C	n	m	${\dot{\varepsilon }}_{0}$ (s^−1^)	*T_r_* (°C)	*T_m_* (°C)
452	457	0.01	0.357	1.1	1	20	604

## Data Availability

Data sharing is not applicable to this article.
